# Cultural Competence and Ethics Among Nurses in Primary Healthcare: Exploring Their Interrelationship and Implications for Care Delivery

**DOI:** 10.3390/healthcare13172117

**Published:** 2025-08-26

**Authors:** Lampros Theodosopoulos, Evangelos C. Fradelos, Aspasia Panagiotou, Foteini Tzavella

**Affiliations:** 1 Department of Nursing, University of Peloponnese, 22131 Tripoli, Greece; aspasi@uop.gr (A.P.); tzavella@uop.gr (F.T.); 2 Laboratory of Clinical Nursing, Department of Nursing, University of Thessaly, 41110 Larissa, Greece; efradelos@uth.gr

**Keywords:** cultural competence, ethics, nursing, transcultural self-efficacy, primary healthcare, ethical decision-making

## Abstract

**Background/Objectives**: Cultural diversity in healthcare settings is rapidly increasing, posing complex ethical and communication challenges for nurses. Competence in navigating cultural differences, alongside ethical sensitivity, is essential to delivering patient-centered care. This study aimed to examine the relationship between nurses’ cultural competence—measured as transcultural self-efficacy—and their knowledge, attitudes, and practices concerning healthcare ethics in primary care settings in Greece. **Methods**: A cross-sectional study was conducted with 492 nurses using validated Greek versions of the Transcultural Self-Efficacy Tool (TSET–Gr) and the Nurses’ Ethics Questionnaire (NEQ–Gr). Descriptive and inferential statistics were used to assess correlations and predictive relationships between demographic variables, cultural self-efficacy subscales, and ethical constructs. **Results**: Nurses demonstrated moderate to high levels of cultural self-efficacy and ethical sensitivity. Affective self-efficacy was the highest-rated subscale and showed strong positive associations with ethical knowledge (r = 0.27, *p* < 0.001) and ethical attitudes (r = 0.23, *p* < 0.001). Multivariable linear regression analysis revealed that higher educational attainment significantly predicted both practical (b = 0.12, *p* = 0.045) and affective self-efficacy (b = 0.15, *p* = 0.002), as well as better ethical knowledge and attitudes. Notably, more years of experience were associated with lower self-perceived cultural competence. Nurses working in multicultural regions reported more favorable ethical orientations. **Conclusions**: Cultural competence, particularly emotional readiness to engage with cultural diversity, is a significant predictor of ethical awareness and behavior in nursing practice. Investment in continuous professional development, education, and supportive work environments is essential for cultivating both ethical sensitivity and culturally responsive care.

## 1. Introduction

### 1.1. Background and Rationale

As the world becomes increasingly interconnected through globalization, societies are becoming more and more culturally diverse. Technological advancements and the digital economy further shape population diversity by enabling remote work and international collaboration, leading to culturally mixed communities [1]. Another significant reason for this diversity is the rapid movement of people across borders due to economic opportunities, conflicts, and climate change that has led to multicultural healthcare environments where patients present varying linguistic, religious, and cultural needs [2]. In primary healthcare, where early diagnosis, treatment, and prevention efforts are critical, the ability of nurses to deliver culturally competent care is essential to ensuring equitable and effective healthcare services [3]. However, navigating cultural differences in healthcare can present ethical dilemmas, particularly when cultural beliefs and traditions conflict with biomedical principles or standard healthcare practices [4].

Cultural competence is increasingly recognized as a fundamental skill in nursing, ensuring that healthcare providers are equipped to understand and respond to the diverse needs of their patients. At its core, cultural competence involves awareness, knowledge, and the ability to apply culturally appropriate communication and interventions in healthcare settings [5]. Meanwhile, nursing ethics serve as a moral compass, guiding healthcare professionals to uphold patient rights, promote equity, and practice non-discriminatory care. However, ethical dilemmas frequently arise when cultural expectations intersect with healthcare policies, particularly in primary healthcare settings, where nurses play a crucial role in decision-making and patient advocacy [6]. Despite the significance of these two concepts, research exploring the direct interrelationship between cultural competence and ethical nursing practice remains limited. This study aims to fill this gap by examining how cultural competence influences ethical decision-making among nurses in primary healthcare or how Knowledge, attitudes and practice of healthcare ethics and law among nurse influences cultural competence.

### 1.2. Cultural Competence in Nursing

Cultural competence in nursing refers to the ability of healthcare providers to effectively engage with patients from diverse backgrounds by acknowledging and respecting their cultural perspectives, values, and traditions. This concept extends beyond language barriers to encompass deeper aspects of cultural sensitivity, including respect for religious beliefs, dietary preferences, and social norms that shape patient health behaviors [7]. Models such as Campinha-Bacote’s model of cultural competence emphasize that cultural competence is not an innate trait but rather a skill that must be cultivated through continuous education, self-awareness, and clinical experience [8].

A growing body of research suggests that culturally competent nursing care is linked to improved patient satisfaction [9], better adherence to treatment [10], and overall enhanced healthcare outcomes [11]. Studies have demonstrated that when nurses possess cultural competence, they are better able to build trust with their patients, minimize misunderstandings, and create a healthcare environment where individuals feel valued and respected [12]. However, a lack of cultural competence can contribute to disparities in healthcare access and quality, as patients from minority backgrounds may feel alienated or misunderstood in clinical settings [13].

Given these findings, integrating cultural competence training into nursing education and professional development programs has become a priority for healthcare organizations worldwide [14]. Several national and international health bodies, including the World Health Organization (WHO), have advocated for cultural competence as a key competency in healthcare, emphasizing its role in eliminating health disparities and improving healthcare accessibility [15,16]. However, while cultural competence is widely recognized as an essential component of nursing practice, its connection to ethical decision-making requires further exploration.

### 1.3. Ethics in Nursing Practice

Ethical principles form the foundation of nursing practice, ensuring that healthcare providers act with integrity, respect, and compassion. The core ethical principles guiding nursing include autonomy, beneficence, non-maleficence, and justice, all of which are designed to protect the rights and dignity of patients [17]. Autonomy, for example, emphasizes a patient’s right to make informed decisions about their healthcare, while justice ensures fair and equitable access to medical resources. However, these principles can sometimes come into conflict with cultural traditions and values, creating complex ethical dilemmas for nurses [18].

One of the most significant ethical challenges faced by nurses in culturally diverse settings involves balancing respect for cultural traditions with adherence to professional ethical guidelines [19]. One of the key ethical challenges in multicultural healthcare settings is the principle of autonomy. In some cultures, family members may make healthcare decisions on behalf of a patient, which can contradict Western bioethical principles that prioritize individual autonomy [20]. Similarly, end-of-life care preferences, religious beliefs regarding medical interventions, and differing attitudes toward gender roles in healthcare can all create ethical conflicts for nursing professionals [21,22,23].

Another key ethical consideration is healthcare equity which refers to the principle of justice. Research has shown that minority and immigrant populations often experience barriers to healthcare, including discrimination, language barriers, and lower-quality care [24]. Nurses, as patient advocates, must navigate these systemic challenges while ensuring that all patients receive equitable treatment regardless of cultural background [25]. This responsibility highlights the importance of cultural competence as a tool for ethical nursing practice, as it enables nurses to recognize and address potential biases while upholding ethical standards.

### 1.4. The Interrelationship Between Cultural Competence and Ethics

The integration of cultural competence and ethical principles is essential for ensuring patient-centered care in today’s multicultural healthcare environments. Culturally competent nurses are better equipped to navigate ethical dilemmas by understanding the cultural contexts that influence patients’ health beliefs and decisions [26]. According to Kuhlmann & Tallman [27] a nurse who understands cultural variations in pain expression is more likely to make ethically sound treatment decisions that align with a patient’s needs, reducing the risk of undertreatment or misinterpretation.

Conversely, a lack of cultural competence can lead to ethical conflicts that compromise patient safety and trust. Miscommunication due to language barriers, for instance, can result in patients receiving inadequate information about their condition or treatment, which violates the ethical principle of informed consent [28]. Similarly, cultural misunderstandings may lead to bias in clinical decision-making, negatively impacting patient outcomes [29]. Nursing education and training programs must emphasize both cultural competence and ethical reasoning to prepare healthcare professionals for the complexities of modern clinical practice.

### 1.5. Study Objectives

This study sought to investigate the relationship between nurses’ cultural competence and their ethical knowledge, attitudes, and practices within the context of primary healthcare delivery in Greece. Cultural competence was assessed through the lens of transcultural self-efficacy, which encompasses cognitive, practical, and affective domains. Ethical competence was explored in terms of knowledge of legal/ethical frameworks, moral attitudes, and responses to common ethical dilemmas in clinical practice.

The general aim was to explore the extent to which cultural competence and ethical awareness co-exist and interrelate among nurses working in public primary healthcare settings.

To operationalize this aim, the study focused on the following specific objectives:

- To assess the levels of transcultural self-efficacy (cognitive, practical, affective) among nurses.
- To evaluate nurses’ ethical knowledge, ethical attitudes, and practices regarding common ethical and legal dilemmas in primary care.
- To examine the associations between demographic/professional characteristics and levels of cultural and ethical competence.
- To investigate the interrelationships among the dimensions of transcultural self-efficacy and the variables related to ethics.
- To identify predictors of transcultural self-efficacy and ethical competence using multivariate analysis.

Based on existing literature, the study tested the following hypotheses:

- Higher educational attainment will be positively associated with greater levels of both cultural and ethical competence.
- Cultural competence and ethical competence will be positively correlated.
- Nurses working in more culturally diverse or ethically demanding settings (e.g., refugee-hosting areas) will demonstrate higher levels of ethical awareness and sensitivity.

By addressing these objectives, this study contributes to the growing body of knowledge on the role of cultural competence in ethical nursing practice. The findings hopefully will provide valuable insights for nursing education, professional development, and healthcare policy, reinforcing the need for an integrated approach to cultural competence and ethics in primary healthcare.

## 2. Materials and Methods

### 2.1. Study Design and Setting

This study employed a cross-sectional design to explore the association between cultural competence and ethical–deontological knowledge, attitudes, and practices among registered nurses working in public primary healthcare settings across mainland and insular Greece. The choice of Greece as the study setting is significant, as the country—and particularly its islands—serves as a major gateway for migrants and refugees into Europe. Nurses in these regions are frequently engaged in delivering care to culturally diverse patient populations, making them an ideal sample for this investigation.

### 2.2. Study Population and Sampling

The study population comprised all registered nurses employed in public primary healthcare units within the jurisdiction of the 2nd Regional Health Authority of Piraeus and the Aegean, an area characterized by significant cultural diversity and a high presence of migrant populations. A census-based sampling strategy was employed rather than a probabilistic sample, with the intention of capturing the full spectrum of professional experiences, cultural interactions, and ethical perspectives in the specific healthcare setting.

Inclusion criteria included being a currently practicing registered nurse in the public primary healthcare sector and having at least one year of professional experience. Nurses working in managerial positions were excluded, as their roles generally do not involve direct patient interaction. Student nurses and professionals working outside the public primary healthcare context were also excluded, as their professional profiles did not align with the objectives of the study.

A total of 737 printed questionnaires were mailed to all eligible healthcare units, accompanied by a cover letter explaining the study purpose and instructions for anonymous return. Of these, 492 were completed and returned, yielding a response rate of 67%. This is considered robust for cross-sectional survey research in healthcare settings and reflects broad engagement from the target population.

Given the census design, traditional statistical power analysis was not applied. However, the final sample size proved adequate for the planned statistical procedures, including multivariate regression models. While this design enhances internal representativeness of the regional nursing workforce, caution should be exercised when generalizing findings beyond the specific geographical and organizational context.

### 2.3. Instruments and Data Collection

Data were collected using a composite questionnaire, combining two validated tools designed to assess cultural competence and ethical knowledge and attitudes and a demographic section. The latter collected socio-demographic and professional information including gender, age, marital status, level of education, years of professional experience, region of employment (island or mainland), and whether the facility was located in a refugee-hosting area.

#### 2.3.1. Transcultural Self-Efficacy Tool (TSET–Gr)

To assess cultural competence, the TSET–Gr [30] was used. This instrument was designed as a diagnostic tool to measure and evaluate nurses’ perceptions of self-efficacy concerning cultural care of patients from diverse backgrounds. The original Transcultural Self-Efficacy Tool (TSET) was developed and tested by Jeffreys and Smodlaka [31,32] and Jeffreys [33]. The third edition of the tool was released by Jeffreys [34]. According to the authors, the TSET is conceptually based on Bandura’s Social Learning Theory and self-efficacy as well as a review of the relevant transcultural nursing literature. This tool comprises 83 items rated on a 10-point Likert scale, where 1 = not at all confident and 10 = completely confident. It is divided into three subscales:

- Cognitive (25 items): Evaluates self-efficacy regarding knowledge of cultural factors influencing nursing care.
- Practical (30 items): Assesses self-efficacy in conducting culturally sensitive nursing interviews. Interview topics include items such as language preferences, religion, discrimination, and attitudes about health and illness.
- Affective (28 items): Measures self-efficacy related to respecting values, attitudes and beliefs concerning cultural awareness, acceptance, appreciation, recognition, and advocacy.

In the present study, internal consistency reliability was excellent, with Cronbach’s alpha coefficients of 0.975 for the cognitive, 0.975 for the practical, and 0.96 for the affective subscale.

#### 2.3.2. Nurses’ Ethics Questionnaire (NEQ–Gr)

To measure knowledge, attitudes, and practices related to healthcare ethics, the Nurses’ Ethics Questionnaire (NEQ–Gr) [35] was used. The original questionnaire was developed and tested by Hariharan et al. [36]. This 28-item instrument includes two sections:

The first section (13 items) evaluates:

- Perceived frequency and clinical impact of common ethical/legal dilemmas in nursing practice;
- Perceived relevance of ethical/legal knowledge in clinical decision-making, including knowledge acquisition pathways;
- Preferred consultation resources when addressing ethical/legal challenges;

Response formats include binary (Yes/No) choices and Likert-type scales.

The second section (15 items) assesses ethical dilemmas commonly encountered in nursing practice, employing a 5-point Likert scale (1 = strongly disagree to 5 = strongly agree). Participants rated their agreement with statements addressing diverse ethical and legal challenges, thereby elucidating their ethical perspectives and bioethical awareness in clinical contexts.

In this study, the Cronbach’s alpha for the 10-item ethical attitudes subscale was 0.65, indicating acceptable internal consistency.

In the interpretation of results, descriptive categories such as “moderate to high levels of self-efficacy” or “positive ethical attitudes” refer to average item scores between 6–10 on the TSET–Gr and 3.5–5 on the NEQ–Gr, respectively. Although no universal cut-off values exist for these tools, these ranges are consistent with previous research using similar instruments in nursing populations.

Permissions for use of both tools were obtained from the authors of the tools, as well as from the authors of the original tools.

### 2.4. Data Collection Procedure

Data collection took place between 20 February 2024, and 31 May 2024. A total of 737 printed questionnaires were grouped into envelopes and mailed to all Public Primary Healthcare Units in the selected area of the 2nd Regional Health Authority of Piraeus and the Aegean. Each package included instructions for anonymous completion and return, along with an informed consent statement. Participation was voluntary, and the return of a completed questionnaire was considered as informed consent to participate in the study. Two reminders were issued during the collection period—one after the second week and another in the final month—to encourage participation and maximize the response rate.

### 2.5. Statistical Analysis

Data analysis was performed using IBM SPSS version 28.0. Categorical variables are presented as absolute and relative frequencies, while continuous variables are presented as mean, standard deviation, median, minimum, and maximum values. The Kolmogorov–Smirnov test was applied to assess the normality of distribution for quantitative variables.

For the bivariate analysis, several statistical tests were applied (independent samples *t*-test, One-Way Analysis of Variance (ANOVA), Pearson’s correlation coefficient, Spearman’s correlation coefficient), depending on the nature of the variables.

To further examine associations and control for confounding variables, multivariable linear regression analysis was performed. In this case, dimensions of cultural competence and ethical outcomes were the dependent variables, while demographic and professional variables were the independent variables (predictors). As we mentioned above, first we performed bivariate analysis. Independent variables that were significantly different (*p* < 0.20) in bivariate analysis were entered into multivariable linear regression models with dimensions of cultural competence and ethical outcomes as the dependent variables. Criteria for entry and removal of variables were based on the likelihood ratio test, with enter and remove limits set at *p* < 0.05 and *p* > 0.10. Multivariate linear regression analyses were applied for the control of each potentially confounding of each statistically significant predictive factor to the others. We present statistically significant predictors for simplicity. In particular, results are expressed as regression coefficients (b), along with 95% confidence intervals and corresponding *p*-values. Statistical significance was set at *p* < 0.05 (two-tailed).

### 2.6. Ethical Considerations

The study was conducted in accordance with the ethical standards of the institutional and national research committees. Ethical approval was granted by the Research Ethics and Deontology Committee of the University of Peloponnese. Further implementation approval was obtained from the Scientific Council of the 2nd Regional Health Authority of Piraeus and the Aegean.

Informed consent was obtained from all participants, and anonymity and confidentiality were rigorously maintained throughout the study.

## 3. Results

A total of 492 registered nurses working in public primary healthcare settings across mainland and insular Greece participated in the study, sharing their views on cultural competence and ethical–deontological attitudes. The results are presented in alignment with the study objectives, covering sociodemographic characteristics, descriptive statistics of cultural competence and ethical knowledge/attitudes, and inferential analyses examining associations among variables.

### 3.1. Participant Demographics

The mean age of the respondents was 42.2 years (range: 20–66, SD = 9.7), with a median age of 42 years. The majority of participants were female (88.7%) and married or in a civil partnership (64.9%).

In terms of educational background, 77.6% held a degree from a Technological Educational Institute (TEI) or University (AEI), 22.4% were graduates of the two-year basic nursing programs, 26.2% had obtained a postgraduate (Master’s) degree, and 0.8% held a doctoral degree (PhD).

Regarding workplace location, 63.6% of participants were employed in mainland units, while 36.4% worked in island-based healthcare units. Notably, 18.1% were employed in areas with a Reception and Identification Center or a closed-controlled facility under the jurisdiction of the Ministry of Migration and Asylum, serving migrant and refugee populations.

The mean years of professional nursing experience was 16 years (SD = 9.9), with a range from 8 months to 40 years and a median of 15 years.

The demographic and professional characteristics of the participants are summarized in Table 1.

### 3.2. Cultural Competence Levels Among Nurses

The Transcultural Self-Efficacy Tool—Greek version (TSET–Gr) demonstrated excellent internal consistency across all subscales. Cronbach’s alpha coefficients were:

- 0.975 for the Cognitive subscale;
- 0.975 for the Practical subscale;
- 0.960 for the Affective subscale.

This indicates a high level of reliability for assessing cultural competence among nurses.

The descriptive statistics for each subscale are presented in Table 2. Higher scores reflect greater self-efficacy in the corresponding domain of cultural competence. Participants reported the highest mean score in the Affective subscale (M = 7.4, SD = 1.3), followed by the Cognitive subscale (M = 7.2, SD = 1.6) and the Practical subscale (M = 6.9, SD = 1.5). In all three domains, the average scores indicate moderate to high levels of perceived transcultural self-efficacy.

An item-level analysis of the Transcultural Self-Efficacy Tool—Greek version (TSET–Gr) offered nuanced insights into specific areas where nurses felt confident or uncertain when delivering culturally responsive care.

In the Cognitive Self-Efficacy subscale, the highest confidence levels were reported in understanding how cultural factors influence care related to hygiene (M = 7.5, SD = 2.1) and health restoration (M = 7.5, SD = 1.9). On the other hand, the lowest confidence levels were observed in items concerning sexuality (M = 7.0, SD = 2.3) and aspects of death, grief, and loss (M = 7.1, SD = 2.3), reflecting some uncertainty when addressing these sensitive and complex areas within culturally diverse contexts.

In the Practical Self-Efficacy subscale, nurses felt most confident when assessing patients’ views on personal space and physical contact (M = 7.4, SD = 1.9) and the role of family in illness (M = 7.1, SD = 1.9). However, lower confidence was reported in engaging with patients on traditional medical practices (M = 6.5, SD = 2.0) and worldviews or life philosophies (M = 6.6, SD = 2.1), highlighting potential challenges in addressing deeper cultural beliefs and traditional health behaviors during clinical interactions.

In the Affective Self-Efficacy subscale, participants expressed strong self-efficacy in being aware of their own biases and limitations (M = 8.4, SD = 1.4) and their cultural heritage and beliefs (M = 8.3, SD = 1.5), as well as in appreciating culturally sensitive care. However, they felt least confident in their awareness of intra-cultural variation within their own culture (M = 5.2, SD = 1.4) and in recognizing the value of traditional home remedies and folk practices (M = 6.0, SD = 2.2). These findings suggest that while affective cultural self-awareness is generally high, there may be gaps in understanding cultural complexity and traditional knowledge systems.

### 3.3. Knowledge and Attitudes of Nurses About Ethics

The Nurses’ Ethics Questionnaire (NEQ–Gr) evaluated nurses’ experiences, knowledge, and attitudes regarding ethical and legal issues in clinical practice. The results are organized into two main sections: (I) perceptions of ethical/legal challenges and resources, and (II) attitudes toward ethical and bioethical dilemmas. Descriptive findings for key domains are summarized below, with corresponding data about some of them, shown in Table 3 and Table 4.

Section I: Perceptions of Ethical/Legal Challenges and Institutional Support

Most nurses reported facing ethical or legal dilemmas regularly, with 33.1% encountering them monthly and 28.9% weekly. Over 40% indicated that such dilemmas moderately complicated their clinical practice. Nearly half the sample reported being at least occasionally required to perform actions that conflicted with their ethical or legal views.

The mean ethical knowledge score was 2.4 (SD = 0.9) out of 4, reflecting moderate awareness of core ethical frameworks such as the Hippocratic Oath, the Nuremberg Code, and the Declaration of Helsinki. Knowledge was primarily acquired through workplace experience (74.2%) and formal education (72.2%), with a smaller proportion attributing their learning to self-directed study (38.8%) (Table 3). Regarding the usefulness of ethical information sources, most nurses found lectures (96.5%) and conferences (95.9%) to be very helpful, followed closely by books (92.7%).

**Table 3:** Ethical/Legal Knowledge of Nurses (N = 492).

Question	Response Category	n	%
Knowledge of Laws Related to Professional Duties			
	None	11	2.2%
	A few	168	34.1%
	Most	232	47.2%
	Unsure	81	16.5%
Importance of Ethical Knowledge in Clinical Practice			
	Not at all	0	0.0%
	Slightly important	3	0.6%
	Moderately important	79	16.1%
	Very important	401	81.5%
	Unsure	9	1.8%
Sources of Ethical/Legal Knowledge	Formal education	355	72.2%
	Work experience	365	74.2%
	Seminars/lectures	145	29.5%
	Personal study	191	38.8%
	Other sources (media/internet)	76	15.4%
Familiarity with Major Ethical Frameworks			
	Hippocratic Oath	457	92.9%
	Nuremberg Code	160	32.5%
	Nursing Code of Ethics	403	81.9%
	Declaration of Helsinki	142	28.9%

When addressing ethical dilemmas, participants most often sought consultation from colleagues (72.8%) and supervisors (67.3%), while one in four (24.6%) also consulted family or friends. For legal challenges, the top choices were lawyers (70.1%), supervisors (52.2%), and colleagues (44.7%).

Most nurses (72.8%) believed that their workplace should have an Ethics Committee. The most widely endorsed functions of such a committee included advising staff on ethical and legal issues (88.6%), upholding institutional ethical standards (84.1%), and guiding administrative decisions related to ethics and policy (71.7%).

Section II: Ethical and Bioethical Attitudes

Participants’ ethical attitudes were assessed using a 10-item scale, which yielded a mean score of 3.8 (SD = 0.4), with a median of 3.7, a minimum of 2.5, and a maximum of 5.0. The Cronbach’s alpha for this scale was 0.650, indicating acceptable internal consistency. Most nurses demonstrated strong support for fundamental ethical principles. The majority agreed that patients should always be informed of medical errors (79.5%), should be actively involved in decision-making about their care (75.6%), and should receive full disclosure of their health condition, even when facing serious illness (73.2%).

The second part of this section explored views on controversial bioethical dilemmas (Table 4). A substantial proportion of nurses (63.0%) opposed the notion that a patient who wishes to die should be assisted, regardless of their illness. On the topic of treatment refusal, 80.7% stated that healthcare professionals should advise patients on the appropriate medical course of action, whereas only 15.7% supported fully respecting the patient’s decision without question. Views on abortion were more divided: 46.5% agreed that a nurse cannot refuse to participate in an abortion when it is legally permitted, 25.0% disagreed, and 28.5% were unsure. In contrast, near consensus was observed on the topic of parental consent for pediatric care, with 79.9% of participants agreeing that children should never be treated without the consent of a parent or guardian, except in emergencies. Finally, just over half (54.2%) disagreed that organ donation should proceed automatically without family consent, while 22.6% supported this policy, and 23.2% remained uncertain.

**Table 4:** Nurses’ Attitudes Toward Common Bioethical Issues (N = 492).

Statement	Strongly Disagree	Disagree		Unsure	Agree		Strongly Agree
A patient who wishes to die should be assisted regardless of illness	23.2%	39.8%		28.9%	6.3%		1.8%
A nurse cannot refuse to participate in an abortion when legally permitted	7.1%	17.9%		28.5%	37.8%		8.7%
Children should not be treated without parental consent (non-emergency)	0.6%	4.1%		15.4%	43.9%		36.0%
Organ donation should occur automatically without family consent	21.5%	32.7%		23.2%	16.1%		6.5%
**Statement**	**Respect the patient’s decision**		**Suggest the right treatment**			**Proceed without consent**	
If a patient refuses transfusion/surgery/therapy, what should the healthcare professional do?	15.7%		80.7%			3.7%	

### 3.4. Correlations Between Cultural Competence and Knowledge and Attitudes of Nurses About Ethics

To examine the interrelationships among demographic, professional, and outcome variables, a series of bivariate correlations and multivariable linear regression analyses were conducted. The independent variables included in the multivariable linear regression models were selected based on their theoretical relevance and statistical significance in the preceding bivariate analyses, in accordance with best practices for cross-sectional study designs. To account for potential confounding effects, multivariate models were adjusted for key sociodemographic and professional characteristics, including age, gender, educational level, years of experience, region of employment (island or mainland), and work placement near Refugee Reception or Closed-Controlled Structures. The focus was on the three subscales of transcultural self-efficacy (cognitive, practical, affective), as well as ethical knowledge and ethical attitudes. The level of statistical significance was set at *p* < 0.05.

Multivariable linear regression analysis indicated that cognitive self-efficacy was significantly and negatively associated with years of professional experience (b = –0.03, 95% CI: –0.04 to –0.01, *p* = 0.001). This suggests that nurses with more years in practice reported lower confidence in their knowledge cultural factors influencing care. No other demographic or professional variable showed a statistically significant association with cognitive self-efficacy.

In terms of practical self-efficacy, two significant predictors emerged. A negative relationship was observed with years of experience (b = –0.02, 95% CI: –0.03 to –0.007, *p* = 0.003), indicating that more experienced nurses expressed less confidence in culturally sensitive communication and patient interaction. Conversely, educational level was positively associated with practical self-efficacy (b = 0.12, 95% CI: 0.002 to 0.24, *p* = 0.045), implying that higher education enhanced perceived competency in conducting culturally sensitive nursing interviews.

Affective self-efficacy, which reflects attitudinal and emotional readiness to provide culturally appropriate care, was significantly predicted by educational level alone (b = 0.15, 95% CI: 0.06 to 0.25, *p* = 0.002). Nurses with higher academic qualifications were more likely to report greater affective self-efficacy, indicating stronger cultural acceptance, empathy, and advocacy.

With respect to ethical outcomes, ethical attitudes were significantly influenced by two factors. First, educational level was positively associated with more favorable ethical attitudes (b = 0.03, 95% CI: 0. 001 to 0.07, *p* = 0.048). Second, working in a region that hosts a Refugee Reception or Closed-Controlled Structure was also a strong predictor (b = 0.18, 95% CI: 0. 09 to 0.28, *p* < 0.001). These findings suggest that both formal education and multicultural work environments enhance nurses’ ethical sensitivity and perspectives in clinical settings.

Ethical knowledge, as measured by awareness of key ethical and legal frameworks, was significantly associated with educational level (b = 0.10, 95% CI: 0.02 to 0.17, *p* = 0.014), further supporting the critical role of academic preparation in the development of ethical competency among nursing professionals.

Pearson’s correlation analysis revealed statistically significant interrelations among some of the study’s primary variables as shown in Table 5. There were strong positive associations between all three subscales of transcultural self-efficacy. Specifically, cognitive and practical self-efficacy were strongly correlated (r = 0.59, *p* < 0.001), as were practical and affective self-efficacy (r = 0.67, *p* < 0.001), and cognitive and affective self-efficacy (r = 0.47, *p* < 0.001).

Beyond internal self-efficacy dimensions, further associations were observed with ethical constructs. Cognitive self-efficacy was positively correlated with ethical knowledge (r = 0.19, *p* < 0.001), while practical self-efficacy was positively associated with both ethical attitudes (r = 0.11, *p* = 0.02) and ethical knowledge (r = 0.17, *p* < 0.001). Likewise, affective self-efficacy correlated positively with ethical attitudes (r = 0.23, *p* < 0.001) and ethical knowledge (r = 0.27, *p* < 0.001). Finally, ethical knowledge and ethical attitudes themselves were also positively related (r = 0.19, *p* < 0.001), indicating a consistent pattern of alignment between nurses’ knowledge and their moral perspectives.

Further analysis of ethical practice variables revealed that cognitive self-efficacy was weakly but significantly associated with the frequency of ethical conflict with colleagues (r = 0.09, *p* = 0.04), suggesting that those more confident in their cultural knowledge may also be more likely to experience interpersonal ethical tensions. Additionally, ethical attitudes were positively correlated with the perceived importance of ethics in clinical decision-making (r = 0.10, *p* = 0.03), indicating that more ethically inclined nurses place greater value on moral considerations in daily care.

Spearman’s correlations showed a range of statistically significant associations between the core variables and participants’ responses to controversial bioethical dilemmas.

Notably, higher cognitive self-efficacy was negatively associated with agreement that nurses must participate in abortion procedures when legally permitted (r = –0.11, *p* = 0.02), as was ethical knowledge (r = –0.13, *p* = 0.004). This suggests that greater ethical awareness and cultural confidence may be linked to stronger endorsement of conscientious objection.

Conversely, nurses with higher practical and affective self-efficacy, and more positive ethical attitudes, were significantly more likely to support the necessity of parental consent for pediatric care, with correlation coefficients of r = 0.11, r = 0.17, and r = 0.21, respectively (*p* < 0.001 in all cases). These findings reflect a shared commitment to safeguarding minors’ rights through appropriate legal and ethical procedures.

Moreover, ethical attitudes were significantly negatively correlated with support for assisted dying (r = –0.13, *p* = 0.004), and with acceptance of organ donation without familial consent (r = –0.14, *p* = 0.002), indicating that stronger ethical stances among nurses may align with more conservative bioethical values in these domains.

**Figure 1:**
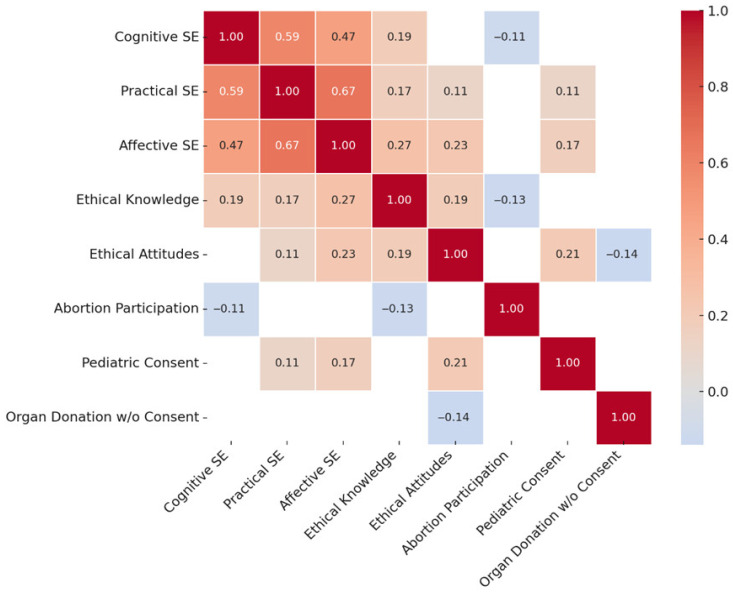
Correlation heatmap showing statistically significant associations between transcultural self-efficacy domains, ethical knowledge, ethical attitudes, and selected bioethical beliefs. Higher correlation coefficients are indicated by darker shading. Positive and negative relationships are shown on a gradient scale.

**Figure 2:**
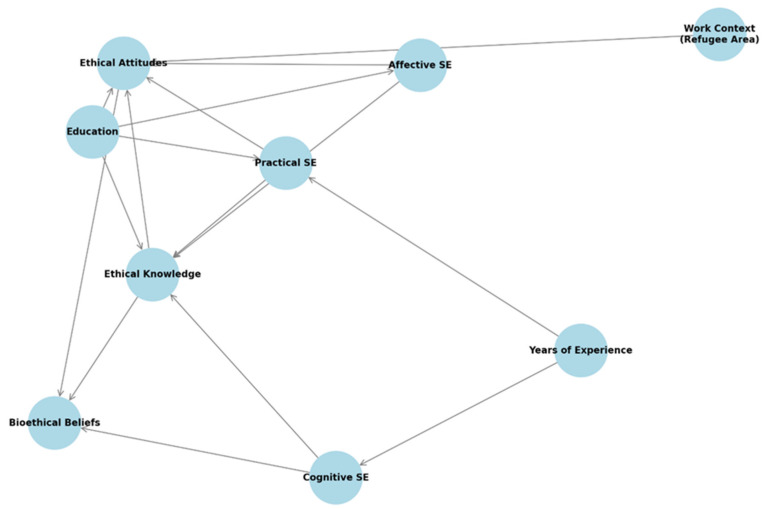
Conceptual diagram summarizing significant predictors and directional interrelationships. Arrows indicate statistically significant multivariate associations among demographic characteristics (education, experience, work context), transcultural self-efficacy subdomains, ethical constructs, and bioethical perspectives.

**Figure 3:**
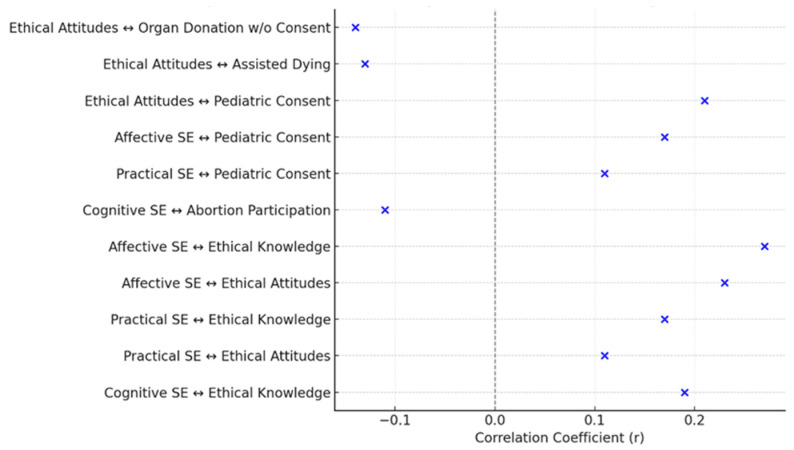
Dot plot showing the strength and direction of statistically significant correlations (Pearson’s or Spearman’s r) between self-efficacy, ethical knowledge and attitudes, and responses to selected bioethical dilemmas. Horizontal position reflects effect size.

To further illustrate the key interrelationships among the study variables, a correlation heatmap (Figure 1), a conceptual diagram of statistically significant predictors and pathways (Figure 2), and a summary plot of effect sizes (Figure 3) are presented below.

## 4. Discussion

This study aimed to explore the interrelationship between cultural competence and ethical decision-making among nurses working in primary healthcare settings across Greece. Specifically, it sought to assess the levels of transcultural self-efficacy among nurses and their knowledge, attitudes, and practices related to ethical and legal issues. It further examined how these dimensions interact and whether specific demographic or professional factors—such as education, years of experience, or multicultural work environments—influence these constructs. In doing so, the study addresses a critical gap in the literature regarding the integrated role of cultural competence and ethical awareness in nursing care delivery.

The findings of this study offer a nuanced view of how cultural competence and ethical understanding coexist and influence each other in the everyday work of nurses in Greece’s primary healthcare system. At the heart of the results is the observation that the three components of transcultural self-efficacy—cognitive, practical, and affective—are not only distinct but also closely interconnected. This supports the idea that being culturally competent is not just about knowing facts or protocols; it also requires having the communication skills and emotional awareness to navigate cultural differences effectively. This pattern echoes what Bandura [37,38] describes in his self-efficacy theory: confidence in one area tends to reinforce competence in other related areas.

This is also consistent with models proposed by scholars such as Papadopoulos [39], who argued that effective transcultural care relies on a balanced development of knowledge, skills, and emotional sensitivity. The strength of the associations among these domains in our study suggests that training interventions aimed at one dimension are likely to enhance the others—a claim supported by previous evaluations of cross-cultural nursing curricula [40]. This multidimensional effect of educational interventions is further emphasized by Leyva-Moral et al. [41], who found that transcultural learning experiences among nursing students lead not only to increased cultural knowledge but also to profound changes in attitudes, self-awareness, and interpersonal skills—highlighting the integrative impact of well-designed curricula.

The inverse relationship we observed between years of clinical experience and cognitive/practical self-efficacy is particularly noteworthy. Interestingly, nurses with more years of professional experience tended to report lower levels of confidence in their cultural knowledge and practical skills. This finding diverges from the assumption that clinical experience naturally enhances all forms of competence. While the relationship might seem surprising, it is likely a reflection of evolving educational content. Nurses trained more recently may have had better exposure to topics like multicultural care and health equity, which have gained prominence in recent decades [6,42,43]. In contrast, nurses who entered the profession earlier may not have had formal training on these issues, highlighting the importance of lifelong learning. This suggests that unless supported by continuous professional development, prolonged clinical exposure may not guarantee increased confidence in cross-cultural communication. On the other hand, education—particularly at higher academic levels—was consistently linked with better self-assessed competence, especially in practical and affective domains. This aligns with existing evidence showing that advanced education contributes not only to clinical skills but also to ethical reasoning and intercultural communication [39,40,44].

Other researchers further support these findings. For instance, Sharma et al. [45] found that healthcare professionals and trainees with more extensive formal education in India reported significantly higher preparedness in addressing the needs of sexual and gender minority patients.

Ethical outcomes in this study were similarly shaped by both education and work environment. Recent studies have shown that structured educational interventions significantly improve nurses’ ethical sensitivity, decision-making, and advocacy behaviors. For instance, Su et al. [46] found that ethics education enhanced nursing students’ capacity for patient advocacy, while Ishihara et al. [47] reported that such training increased nurses’ moral efficacy and confidence in managing ethical challenges in acute care settings. Milliken and Grace [48] emphasize that when nursing curricula intentionally include ethics as an integral component, nurses are more likely to engage thoughtfully with ethical issues and act with greater moral clarity in their professional roles. These findings support the view that ethical competence is not solely intuitive but can be cultivated through targeted educational strategies. Regarding the effect of the work environment, we found that nurses working in refugee-hosting or multicultural areas showed stronger ethical attitudes. Exposure to diverse populations may challenge practitioners to think more deeply about values, fairness, and patient rights. Kotrotsiou et al. [49], similarly observed that Greek nursing students demonstrated higher levels of cultural competence when interacting with patients from various cultural backgrounds, indicating that such exposure enhances nurses’ ethical awareness and sensitivity. Ünsal et al. [50] came to a similar conclusion in their study of Turkish nurses caring for displaced populations after natural disasters, where navigating linguistic and religious differences required a blend of emotional intelligence and ethical reasoning.

What stood out particularly was the strong link between affective self-efficacy—the ability to emotionally engage with patients from different backgrounds—and ethical sensitivity. Nurses who felt more confident about their cultural empathy and open-mindedness were also more likely to demonstrate strong ethical awareness. This suggests that empathy and cultural openness may serve as important bridges between competence and moral action [42]. Affective empathy plays a crucial role in the moral reasoning and ethical decision-making processes of healthcare providers, especially in intercultural contexts. Studies have shown that healthcare professionals who exhibit higher levels of affective empathy are better equipped to navigate complex moral dilemmas and foster effective communication across diverse cultural settings. This ability is paramount in healthcare, where understanding patients’ emotional states can significantly improve patient-provider interactions and overall health outcomes. Jeon and Choi [51] discuss the importance of empathy in healthcare settings, emphasizing that the emotional aspect of empathy, particularly affective empathy, enables practitioners to connect with patients on a deeper psychological level. They argue that while cognitive empathy involves understanding another’s feelings intellectually, affective empathy allows healthcare providers to share and resonate with patients’ emotional experiences. This emotional connection is crucial in fostering patient-centered care, which can enhance patient satisfaction and treatment adherence.

The results also shed light on how cultural and ethical orientations shape nurses’ opinions on controversial bioethical issues. For instance, nurses with higher cognitive self-efficacy and greater knowledge of ethical standards were less likely to support mandatory involvement in abortion procedures, perhaps reflecting a stronger alignment with professional autonomy and the right to conscientious objection. This aligns with the findings of Krawutschke et al. [52] who supports that nurses who object to abortion often see mandatory referrals as complicity in actions they morally oppose, thereby reinforcing their alignment with professional autonomy. Conversely, those with stronger affective and practical cultural competence tended to support parental consent in pediatric care—emphasizing the importance of family involvement and legal safeguards in vulnerable situations. Guarda-Rodrigues [53] also discusses how culturally competent practices promote parental empowerment and enhance the quality of nursing care.

In more ethically complex areas such as assisted dying and organ donation without consent, those with higher ethical attitudes expressed more cautious views, opposing both practices. These positions may reflect an emphasis on informed consent, patient dignity, and moral responsibility, which are central values in nursing ethics globally [54]. Similarly, a 2023 study by Dörmann et al. [55] explored professional nursing views on assisted suicide in Germany. The study highlighted that nurses’ attitudes are shaped by their ability to understand patients’ wishes, personal values, and ethical concerns, leading many to adopt a cautious stance toward assisted suicide.

Additionally, our results contribute to the literature on moral distress and the ethical climate in healthcare institutions. The correlation between cognitive self-efficacy and conflicts with colleagues over ethical issues is an important area of study, especially in ethically diverse environments. Nurses, as ethically aware practitioners, may experience increased tension within rigid or homogeneous teams. This phenomenon highlights how divergent ethical perspectives can create friction, particularly in settings that lack cultural competence alongside ethical awareness. These conflicts may not indicate a deficiency, but rather a heightened moral awareness—a theme that warrants further investigation. Ethical leadership fosters an environment where ethical concerns can be voiced without fear of reprisal. Elhihi et al. [56] found that ethical leadership significantly impacted nurses’ error reporting behavior and moral courage. Their study demonstrated that moral courage partially mediated the relationship between ethical leadership and error reporting, suggesting that leaders promoting an ethical culture can help mitigate conflicts arising from differing ethical viewpoints. This implies that teams lacking strong ethical leadership may struggle with navigating conflicts effectively.

In discussing the relationship between cultural competence, and ethical sensitivity, some recent studies provide findings that warrant careful consideration. While there is a broad consensus in the literature regarding the positive association between cultural competence and ethical outcomes, contrasting evidence exists suggesting that this relationship may not be as robust as often found. Henderson et al. argue that cultural competence extends beyond basic cultural knowledge and skills, emphasizing the importance of developing deeper moral reasoning in healthcare practitioners through engagement with real ethical dilemmas [57]. Their perspective implies that while cultural competence can enhance interpersonal interactions in diverse settings, it does not necessarily correlate with improved ethical sensitivity. This indicates a potential disconnect where practitioners may demonstrate cultural competence without an equivalent enhancement in ethical decision-making abilities. In line with this view, Nair and Adetayo highlight the need for systemic improvements in cultural competence to effectively address healthcare disparities. They contend that although enhanced cultural competence can improve patient care experiences, it does not inherently address the reflective nature of ethical judgments required within clinical practice [58]. Consequently, the correlation between self-efficacy in cultural competence and ethical sensitivity appears to lack direct causation, possibly relying instead on broader structural changes in healthcare delivery and ethics training. Koskenvuori et al. conducted a scoping review which reinforces that ethical competence consists of various elements, including ethical sensitivity, ethical knowledge, and decision-making skills [59]. The study underscores the complexity of ethical competence, which cannot be fully developed through cultural competence training alone. It also posits that professional experiences and reflective practices significantly enhance ethical competence, further complicating the assumption that cultural competence directly improves ethical sensitivity. Moreover, Pettersson et al. conducted qualitative research among healthcare professionals that indicated the importance of regular dialogues on ethics in cultivating a culture of ethical competence [60]. This finding suggests that ethical knowledge and sensitivity may be more effectively fostered through discussion and interpersonal interactions rather than solely through competency training. The focus on dialogue as a mechanism for developing ethical skills implies that exposure to cultural competence education alone may be insufficient for instilling ethical attitudes in practitioners. Kandemir and Yüksel also found negligible links between cultural competence and moral sensitivity, noting the role of other factors, such as resilience, as potential mediators in this relationship, suggesting a more nuanced understanding of ethical practice in nursing contexts [61]. In conclusion, the literature also presents conflicting perspectives on the relationship between cultural competence and ethical sensitivity. This indicates that complex interactions influenced by a range of systemic, experiential, and communicative factors in healthcare settings might exist. Future research should aim to clarify the constructs involved and explore how cultural competence can ideally intertwine with ethical practice.

## 5. Implications for Nursing Practice

This study highlights the ongoing need for structured and continuous professional development in cultural competence, especially in primary care settings where nurses encounter diverse populations. Notably, the finding that longer professional experience was linked to lower cognitive and practical self-efficacy suggests that experiential learning alone may not suffice to sustain culturally responsive practice. Thus, periodic, targeted training in cultural competence, bias awareness, and transcultural communication is essential throughout a nurse’s career.

Healthcare institutions should actively create supportive environments that embed ethical and culturally sensitive care into daily practice. This could include establishing clear protocols for managing cross-cultural situations, promoting case-based ethics discussions during team meetings, and ensuring that clinical supervision addresses both ethical reasoning and cultural awareness. Ethics committees can also serve as practical resources for front-line staff, offering guidance on complex dilemmas in real-time. Additionally, it is recommended that institutions adopt standardized tools—such as the Transcultural Self-Efficacy Tool (TSET) and validated ethics knowledge assessments—for regular evaluation of nurses’ competencies. These assessments can serve both formative and summative purposes, guiding targeted educational interventions and policy development.

In nursing education, there is a strong case for integrating dedicated modules on cultural competence and healthcare ethics into undergraduate and postgraduate curricula. Training should go beyond theoretical instruction to include simulation, interprofessional role-playing, and experiential reflection. This alignment between education, clinical settings, and continuing professional development will help equip nurses not only to deliver equitable and ethical care but also to act as advocates for vulnerable or marginalized patient groups.

Ultimately, fostering ethical and culturally competent nursing requires an ecosystem approach—one that connects policy, practice, and education in meaningful and sustainable ways.

## 6. Limitations of the Study

This study has a few limitations that warrant consideration. First, the use of self-reported questionnaires may have introduced social desirability bias, whereby participants may have portrayed themselves as more culturally competent or ethically aware than they truly are in practice. While validated tools such as the TSET–Gr and NEQ–Gr were used, these instruments assess perceptions rather than observable behaviors, potentially limiting the objectivity of the findings.

Second, the study population consisted exclusively of nurses working in primary healthcare settings in Greece, which restricts the generalizability of the findings to other settings, professions, or cultural contexts. Ethical and cultural practices are shaped by national policies, societal values, and workplace norms, which may differ considerably in other countries or sectors of care.

Third, the study did not assess actual clinical behavior or outcomes, focusing instead on perceived self-efficacy and attitudes. As such, it cannot confirm whether higher self-reported competence translates into culturally sensitive or ethically sound clinical actions.

Finally, the study did not collect data on the personal migration background of the participants, which may have offered additional insights into how personal cultural experiences influence transcultural self-efficacy and ethical perspectives.

## 7. Conclusions

This study provides new insight into the complex relationship between cultural competence and ethical sensitivity among nurses in primary healthcare settings. By assessing transcultural self-efficacy alongside ethical knowledge, attitudes, and practices, it highlights how affective self-efficacy is a key driver of ethical awareness. Furthermore, educational attainment emerged as a consistent predictor of both ethical and cultural competence, underscoring the value of academic preparation in fostering responsive, patient-centered care.

What sets this study apart is its focus on nurses working in public primary care settings, including culturally diverse and migrant-rich regions—an area that remains underexplored in the literature. The findings emphasize the need for integrated, targeted training programs that simultaneously develop cultural competence and ethical reasoning skills.

In light of global healthcare challenges and increasing diversity, the ability of nurses to respond ethically and culturally appropriately is not just a matter of professional excellence, but a cornerstone of equitable and safe care. This research calls for system-level investments in education, institutional support, and policy reforms to ensure that nurses are fully equipped to meet the evolving ethical and cultural demands of their practice.

## 8. Recommendations for Future Research

Building on the insights gained from this study, future research should further explore the development and integration of cultural competence and ethical sensitivity in nursing practice. Longitudinal designs are encouraged to examine how these competencies evolve over time and in response to professional experience, targeted training, or institutional support.

Qualitative approaches, including interviews and focus groups, may provide richer understanding of nurses’ lived experiences when navigating ethically and culturally complex situations. Such methodologies could illuminate underlying values, challenges, and strategies that may not be fully captured through quantitative tools.

Cross-cultural comparative studies across diverse healthcare systems would also be beneficial. These could help determine how sociocultural contexts influence the interplay between ethical reasoning and culturally competent care, and whether similar trends hold across international settings.

Additionally, future research should consider how personal factors—such as a healthcare professional’s own migration background or exposure to multicultural environments—shape attitudes and self-efficacy in delivering equitable, respectful care.

Expanding the research base in these directions will not only validate current findings but also guide the development of more responsive and inclusive nursing education and practice frameworks.

## Figures and Tables

**Table 1:** Demographic and Professional Characteristics of Participants (N = 492).

Socio-Demographic and Professional Variables		n	%
Gender	Male	55	11.3%
	Female	433	88.7%
Age (years)	Mean/Standard Deviation (SD)	42.2	9.7
Marital Status	Single	127	25.9%
	Married	293	59.8%
	In civil partnership	25	5.1%
	Divorced	39	8.0%
	Widowed	6	1.2%
Education Level	Two-year program	110	22.4%
	TEI degree	199	40.4%
	AEI degree	50	10.2%
	Master’s (MSc)	129	26.2%
	Doctorate (PhD)	4	0.8%
Work Unit Location	Mainland	313	63.6%
	Island-based	179	36.4%
Years of Experience	Mean/Standard Deviation (SD)	16.0	9.9
Presence of Migrant Reception or Closed Controlled Center	Yes	89	18.1%
	No	403	81.9%

**Table 2:** Descriptive Statistics for Transcultural Self-Efficacy (TSET–Gr).

Subscale	Mean	SD	Median	Min	Max
Cognitive	7.2	1.6	7.4	1.8	10
Practical	6.9	1.5	7.0	2.8	10
Affective	7.4	1.3	7.4	2.8	10

**Table 5:** Correlations Between Core Study Variables.

	Practical Self-Efficacy		Affective Self-Efficacy		Ethical Attitudes		Ethical Knowledge	
	Pearson’s r	*p*-Value	Pearson’s r	*p*-Value	Pearson’s r	*p*-Value	Pearson’s r	*p*-Value
Cognitive Self-Efficacy	0.59	<0.001	0.47	<0.001	0.03	0.54	0.19	<0.001
Practical Self-Efficacy			0.67	<0.001	0.11	0.02	0.17	<0.001
Affective Self-Efficacy					0.23	<0.001	0.27	<0.001
Ethical Attitudes							0.19	<0.001

## Data Availability

The datasets presented in this article are not readily available due to technical/time limitations. Requests to access the datasets should be directed to L.T.
