# Correction: Theodosopoulos et al. Cultural Competence and Ethics Among Nurses in Primary Healthcare: Exploring Their Interrelationship and Implications for Care Delivery. *Healthcare* 2025, *13*, 2117

**DOI:** 10.3390/healthcare13222983

**Published:** 2025-11-20

**Authors:** Lampros Theodosopoulos, Evangelos C. Fradelos, Aspasia Panagiotou, Foteini Tzavella

**Affiliations:** 1 Department of Nursing, University of Peloponnese, 22131 Tripoli, Greece; aspasi@uop.gr (A.P.); tzavella@uop.gr (F.T.); 2 Laboratory of Clinical Nursing, Department of Nursing, University of Thessaly, 41110 Larissa, Greece; efradelos@uth.gr

## Text Correction

A discrepancy has been identified between the main text and the back matter of the original publication [1], regarding ethical approvals. While the main text mentions that Institutional Review Board approval and informed consent were obtained, the corresponding statements in the back matter are marked as “Not applicable”.

A correction has been made to the Institutional Review Board Statement and Informed Consent Statement. The corrected statements appear below.

**Institutional Review Board Statement:** The study was conducted in accordance with the ethical standards of the institutional and national research committees. Ethical approval was granted by the Research Ethics and Deontology Committee of the University of Peloponnese, protocol code: 7360; date of approval: 3 April 2023. Further approval for this research was obtained from the Scientific Council of the 2nd Regional Health Authority of Piraeus and the Aegean, protocol code: 25025; date of approval: 21 April 2023.
**Informed Consent Statement:** Informed consent was obtained from all subjects involved in the study.

The authors state that the scientific conclusions are unaffected. This correction was approved by the Academic Editor. The original publication has also been updated.
